# Supplementary figures and images for: Fat tissue regulates the pathogenesis and severity of cardiomyopathy in murine chagas disease
Source: PLoS Negl Trop Dis. 2021 Apr 7;15(4):e0008964. doi: 10.1371/journal.pntd.0008964 (PMC8055007; doi:10.1371/journal.pntd.0008964)

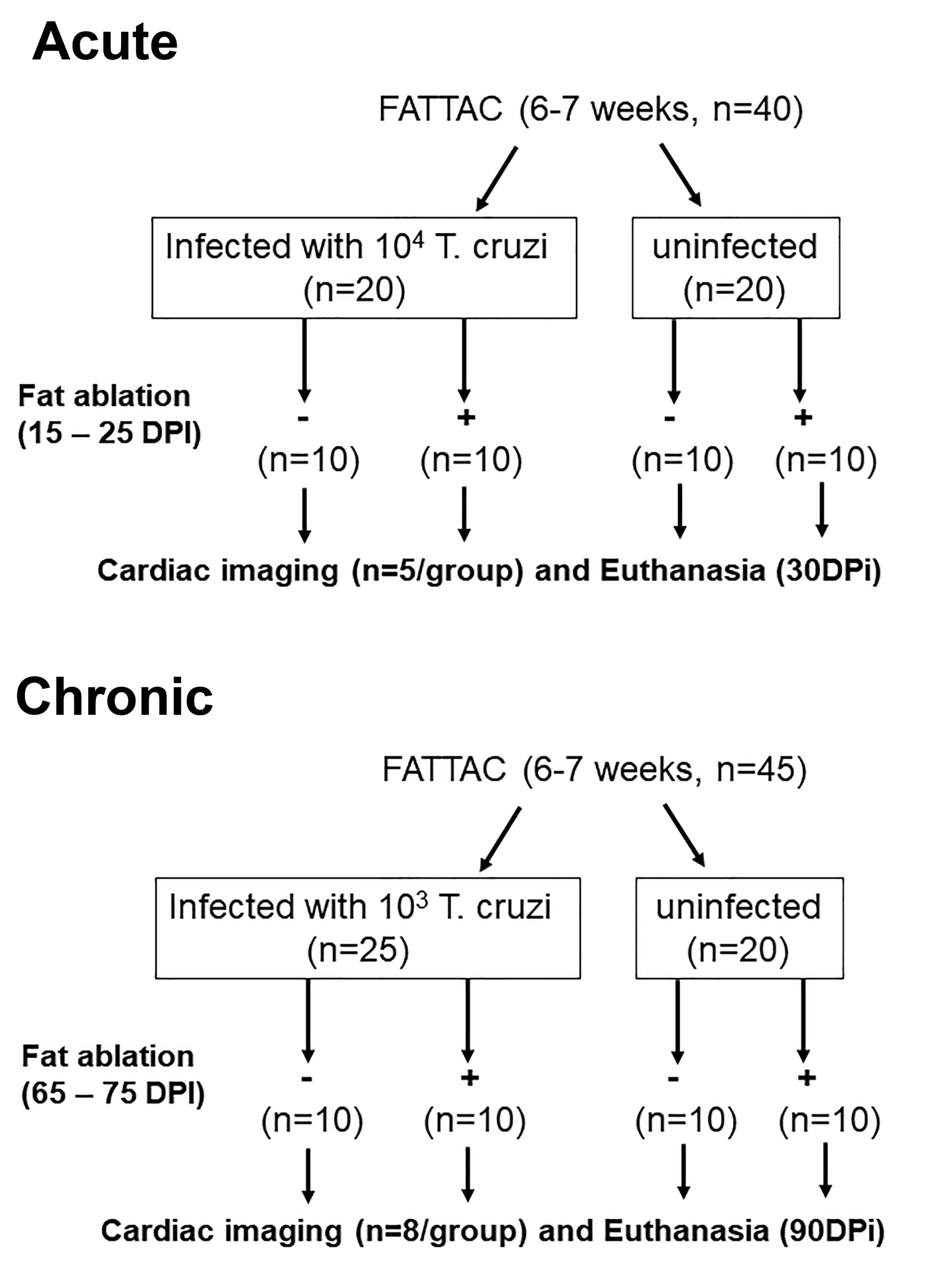

Supplement: S1 Fig — Schematic representation of the experimental design of the acute (a) and chronic (b) induction of disease in mice. (TIF) [file pntd.0008964.s001.tif]

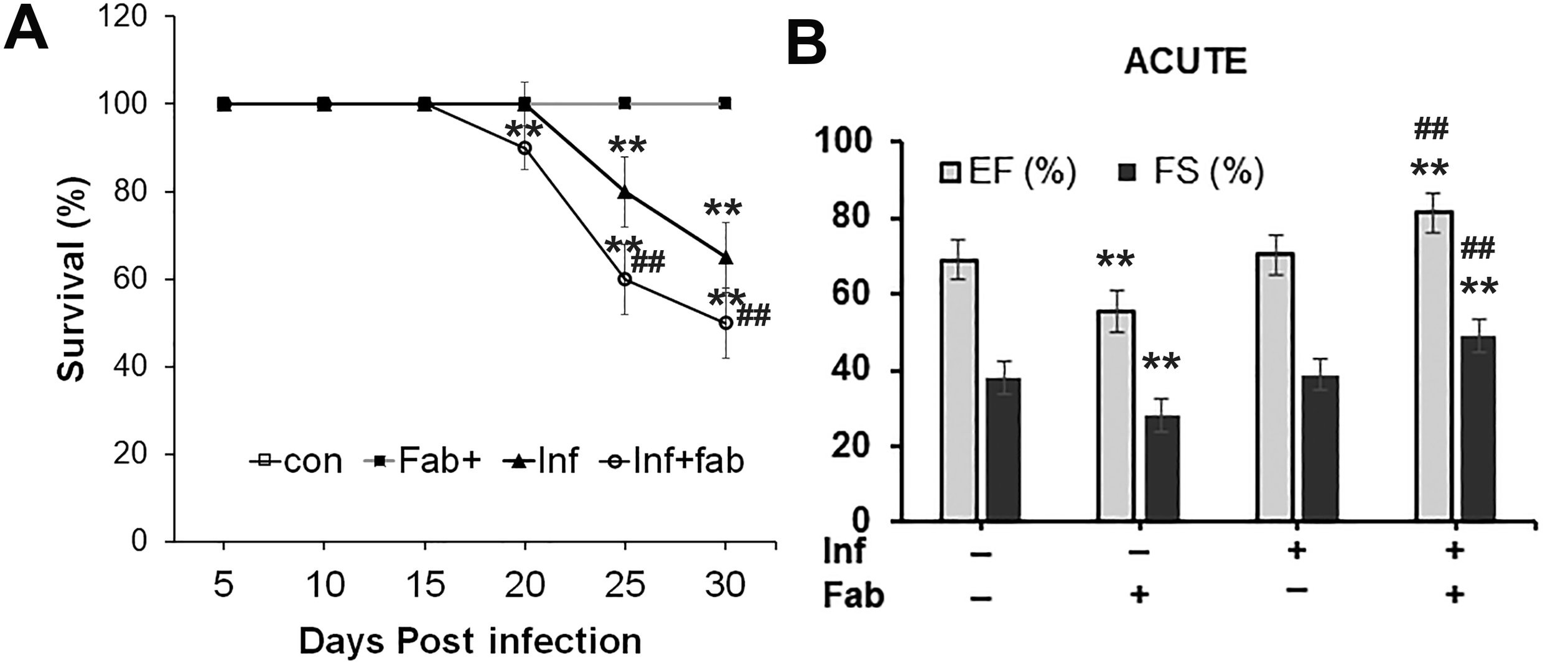

Supplement: S2 Fig — A. Survival plot of acute T. cruzi infected mice with and without fat ablation. B. Change in ejection fraction (EF) and fractional shortening (FS) analyzed by cardiac imaging. *p≤0.05, **p≤0.01 or ***p≤0.001 compared with uninfected fat-unablated. #p≤0.05, ##p≤0.01 or ###p≤0.001 compared with infected fat-unablated. (TIF) [file pntd.0008964.s002.tif]

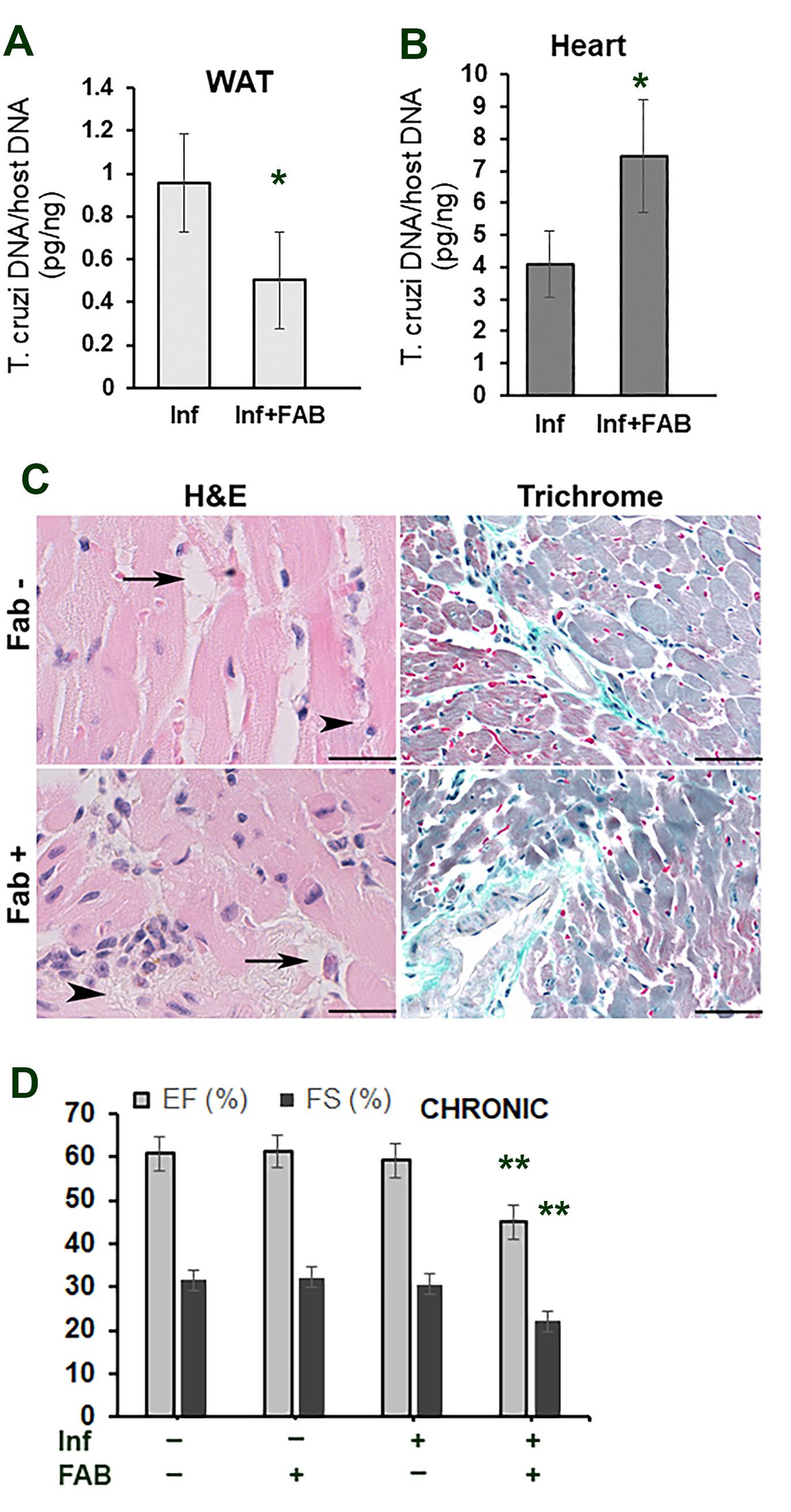

Supplement: S3 Fig — A. Assessment of the parasite load by quantitative PCR in adipose tissue of infected (Inf) and infected fat-ablated (Inf+FAB) mice, *P≤0.05. B. Assessment of the parasite load by quantitative PCR in the hearts of infected (Inf) and infected fat-ablated (Inf+FAB) mice, *P≤0.05. C. Hematoxylin and eosin (H&E) and masson trichrome staining of hearts in indicated mice (infected or uninfected mice, fat-ablated (Fab+) or fat-unablated (Fab-). Presence of lipid macro-lipid droplets (black arrow) and micro-lipid granules (black arrowhead). Bar = 100 μm, 20x magnification. D. Change in ejection fraction (EF) and fractional shortening (FS) analyzed by cardiac imaging. **p≤0.01 compared with uninfected fat-unablated. (TIF) [file pntd.0008964.s003.tif]
