# Supplementary material for: Fat tissue regulates the pathogenesis and severity of cardiomyopathy in murine chagas disease
Source: PLoS Negl Trop Dis. 2021 Apr 7;15(4):e0008964. doi: 10.1371/journal.pntd.0008964 (PMC8055007; doi:10.1371/journal.pntd.0008964)
Supplement: Attachment — Submitted filename: Rebuttal Letter.doc [file pntd.0008964.s004.doc]

03/03/2021

Editor,

Plos Neglected Tropical Diseases Journal

We thank the reviewers for their evaluation and comments. The reviewers both agreed that this manuscript presents interesting data regarding adipose tissue and chronic Chagas disease outcome but expressed minor concerns regarding the parasite load and cardiac functional analysis. We have addressed the concerns of the reviewers in our revised manuscript. The reviewers’ comments have further strengthened our work and clarified our results. A detailed response to the reviewers' suggested modifications and corrections follows. The reviewers’ comments are in italic font 10 text.

**Reviewer# 1’s Comments and Authors’ Response**

***Comment*:** *I find that supporting figures 2A and B have to be figure 1 in the main text, as they provide much more information than current figure 1 alone. Are the survival curves provided in figure 2C statistically different? To facilitate readers' analysis of figures, I suggest that the * and # meaning be included in the figure legends.*

**Response:** As suggested by the reviewer, we included Figure 2B in Figure 1 in the main text. Since the Supporting Figure 2A was similar (except the magnification), we did not include this image in Figure 1 and Supporting Figure 2A was removed in the revised manuscript. We included P values to indicate statistical differences for the survival curves in Supplemental Figure 2C (now, Supplemental Figure 2A). We included the meaning of “* and #” in the figure legends as suggested.

***Comment:*** *The results presented in figure 1 and supplementary are consistent with the overall objectives. However, the authors’ claim that there is “increased cardiac cell death, increased parasite load increased cardiomyopathy and increased mortality in T. cruzi infected mice” deserves further confirmation. Specifically, how was cardiac death determined? Or it arises from the histological appreciation? Supporting figure 2C suggests increased mortality in the FAB+, infected mice, but was it statistically different? The Internal Ventricular Diameter was the only cardiac parameter that showed significant changes?*

**Response:** Cardiac cell death was assessed based on the H&E staining demonstrating characteristic findings of cell death. As suggested, we have removed the words “cardiac cell death” from the main text. Supporting figure 2C (now Supporting figure 2A) has been updated. The statistical data is included in the revised figures. We also included the values for the ejection fractions and fractional shortenings for uninfected mice and other groups in Supporting figure 2B.

***Comment*:** *During the chronic or indeterminate phase, the parasite load in adipose tissue was evaluated? How the adipocyte cell death was evaluated? Authors state in line 296 that “These results suggest that persistence of infiltrated immune cells in adipose tissue alters adipose tissue physiology by causing an imbalance between adipogenesis and lipolysis”. How can be concluded that immune cell infiltration is the direct consequence of the alterations of adipogenesis and lipolysis? Probably a most straightforward conclusion is that parasite presence (in adipose tissue or elsewhere) might be the driving force for this phenomenon.*

**Response**: We have revised the manuscript to include parasite load in the adipose tissue data (Supplemental figure 3A). Adipocyte cell death was assessed based on the histological (H&E sections) data and the immunoblot data (caspase3 and BNIP expression for apoptosis and necrosis, respectively). As suggested we revised the result and discussion sections and included a sentence stating that “the presence of parasites increases infiltration of immune cells and induced adipocyte cell death via necrosis and apoptosis in adipose tissue during the indeterminate/early chronic infection in *T. cruzi* infected mice”.

***Comment*:** *Those questions are answered in further paragraphs. Thus, I suggest to include the preliminary conclusions in lines 258 and 296 in the discussion section. The same applies to the statement in line 320. In this respect, the direct relationship between adipocyte dysfunction and immune cells' infiltration is not apparent since, in FAB+ mice, this indicator is not increased. The relationship between infection and the presence of cell death markers, especially necrosis, is clearer (however, it would be desirable to verify the parasitic load in adipose tissue to confirm this relationship). It is not ruled out that the ablation itself is the cause of the activation of caspase 3, since the mice submitted to this procedure also present elevated levels of cleaved caspase 3.*

**Response**: In the revised manuscript we have added the parasite load data, which are presented in supplemental Fig. 2A. We also included the preliminary conclusions in lines 258 and 296 (previous version) in the discussion section (marked in red in the revised manuscript).

***Comment*:** *If the authors performed doppler echocardiography, why do they only present the diameters of the chambers? What happened to, for example, the ejection fraction and other parameters of heart function? Were they affected enough to support the morphological alterations shown.*

**Response**: In the revised manuscript we included the doppler analysis data (ejection fractions and fractional shortenings) in the results section as well as in the supplemental figures (2A and 3D). They support the conclusions of the manuscript.

***Comment*:** *Finally, why not to show the evidence for the fibrosis involvement in the infected hearts, at least as part of a support figure.*

**Response**: We had included the histological data (Masson staining) in support of the fibrosis in the infected hearts but wrongly labeled as “Acute infection”. We apologize for this typographical error which has been corrected in the revision. In the revised manuscript we have updated Supplemental Figure 3C to provide this data.

**Reviewer#2’s Comments and Authors’ Response**

***Comment*:** *Data on how authors confirmed fat ablation levels should be added in supp. fig 1*

**Response**: An approximately 80% loss in body fat in fat-ablated mice was observed during dissection compared to normal uninfected fat-unablated mice. We did not take the pictures of our murine dissections and cannot, therefore, provide pictures of these exact mice to illustrate the loss of visceral fat in these fat-ablated mice. However, we have previously published a manuscript (https://doi.org/10.1128/mBio.02771-18) that clearly demonstrated loss of body fat in fat-ablated mice and contains pictures documenting this loss. In the revised manuscript we have included this reference in the main text.

***Comment*:** *Sometimes authors make a general conclusion statement of results. Example: after describing results from Fig 1/2, supplement fig1/2, authors conclude changes in many parameters with infection and ablation. While figures present many data points in support of conclusion, cell death and cardiomyopathy are not discussed in data/results. If a decline in LV and increase in RV diameter is considered cardiomyopathy then it should be defined before conclusion.*

**Response**: In the revised manuscript we have discussed in greater detail the effect of fat-ablation on the development of bi-ventricular cardiomyopathy. We have also included additional cardiac functional analysis data to support our earlier conclusion. We have also more carefully defined cardiomyopathy in the revised manuscript.

***Comment*:** *It will be nice if authors can substantiate H&E findings with oil red O or PAS*

**Response**: We used tissue samples embedded in paraffin for histology. We are unable to perform lipid staining analysis in such paraffin embedded tissue sections and unfortunately, we do not have the frozen sections that could be used for oil red O or similar staining. To that end, obtaining this data would be nice, but would require replication of the entire experiment. The western blots demonstrating the increased levels of perilipin (a marker of lipid droplets) in the hearts of infected mice do support the histological analysis data.

We believe by addressing the reviewers’ comments and including more data we have significantly improved this manuscript and that the manuscript is now ready for publication in PlosNTD. The changes made to the manuscript are marked in red font. In the event of publication, we assign copyright in accordance with the instructions to authors.
